# A Rapid and Delicate Method of Detecting Bile Pigment in Urine

**Published:** 1910-09-10

**Authors:** 


### Pathology.

### A RAPID AND DELICATE METHOD OF DETECTING BILE PIGMENT IN URINE.

The best known methods of detecting bile pigments in the urine depend upon the fact that oxidation leads to the production of pigments of different colours; the commonest is that with fuming nitric acid—Gmelin's test. It is well enough known, however, that even in cases of distinct jaundice it may be difficult to get a positive reaction for bile pigments in the urine, and if this is so in patients who are already known to be jaundiced it is still more likely to be so in those slighter cases in which incipient jaundice is suspected but in which there is some doubt. Macadie has described a method of detecting them which is both rapid and more delicate than most other tests. It depends, like most others, on the extraction of bilirubin, and the production of a series of colours. It has the advantage that the amount of oxidation may be regulated and prevented from going so far as to pass through the green stage of biliverdin to the yellow or indeterminate stage of choletelin. About 10 c.c. of urine is acidulated with acetic acid, shaken up well, and to it is added enough of a clear saturated solution of calcium chloride to precipitate the bulk of the urates. The specimen is centrifugalised well, the supernatant liquid is decanted from the sediment, the latter is rinsed with a few drops of water, which is again decanted off and the precipitate left as well drained as possible. The greater part of the bile pigment that was present in the 10 cubic centimetres of urine has been carried down by the precipitated urates. To the latter 5 or 6 cubic centimetres of Macadie's reagent are now added; this consists of one part of hydrochloric acid of specific gravity 1.16 and three parts of rectified spirit of wine. On stirring with a glass rod the urate precipitate dissolves to a more or less clear solution on to the surface of which five or six drops of nitric acid of specific gravity 1.12 are allowed to trickle down the side of the tube. The liquid rapidly assumes a series of colours precisely similar to that of Gmelin's test. At the bottom of the liquid and next to the nitric acid is a yellow layer, above that a wine-red layer, above that a blue layer, above that a bluish-green layer, and above that a green layer. Care should be taken not to shake up the liquid. When bile pigment is present in any quantity the appearance is almost like that of a spectrum. The layers of different colours are not in such close proximity as they are in Gmelin's test, and Macadie states they are therefore much more easily recognised. In doubtful cases, especially when the urine is being tested in a laboratory, the traces of bile pigment from a pint of urine can be collected in quite a small urate precipitate, and this makes the test a very delicate one. With the aid of a centrifugal machine the procedure can be carried out in less than five minutes, and it is not influenced by urobilin, blood pigments, or indican.

The danger of misinterpreting the brown colour produced when the nitric acid is employed is considerable in practice, and the importance of avoiding this source of error is great. The only difficulty that might arise in connection with Macadie's test would be if calcium chloride did not give a precipitate of urates. This must be a rare occurrence, but when it arises one drop of caustic soda solution may be added to the mixture of calcium chloride and urine so as to get a phosphatic instead of a uratic precipitate. The process may then be continued in precisely the same manner as above and the reaction obtained as before.

